# Medications and traffic accidents involving older drivers: do Spanish primary healthcare physicians know enough?

**DOI:** 10.1186/s12877-023-04316-z

**Published:** 2023-10-17

**Authors:** Eladio Jiménez-Mejías, Fátima Ruiz-Rodríguez, Luis Miguel Martín-de los Reyes, José Herrero-Rubí, Mario Rivera-Izquierdo, Virginia Martínez-Ruiz, Pablo Lardelli-Claret

**Affiliations:** 1 Department of Preventive Medicine and Public Health, School of Medicine, University of Granada, Avenida de la Investigación 11, Edificio A, 8ª planta, Granada, 18016 Spain; 2 Centros de Investigación Biomédica en Red de Epidemiología y Salud Pública (CIBERESP), Madrid, Spain; 3 Instituto de Investigación Biosanitaria de Granada (ibs.GRANADA), Granada, Spain; 4 Chair SEMERGEN-UGR of Teaching and Research in Family Medicine, University of Granada, Granada, Spain; 5 Doctorate Program in Clinical Medicine and Public Health, University of Granada, Granada, Spain

**Keywords:** Traffic accidents, Older drivers, Medications, Prevention, Family doctors, Primary healthcare physicians

## Abstract

**Background:**

Our aim was to evaluate Spanish family doctors’ knowledge about medications that increase the risk of traffic accidents involving older drivers, and to obtain data about the involvement of family doctors in accident prevention activities and the associations between these factors and their demographic and workplace characteristics.

**Methods:**

A cross-sectional study of 1888 family doctors throughout Spain was carried out from 2016 to 2018. Participants completed a previously validated self-administered questionnaire that explored whether family doctors distinguished between medications associated with a high or low risk of involvement in a traffic accident, investigated the appropriateness of advice given to older patients, and physicians’ involvement in preventive activities. Multiple regression models were used to estimate the adjusted association of these variables with each other and with characteristics of family doctors in the sample.

**Results:**

On a scale of 1 (never or hardly ever) to 4 (always), the indexes constructed to evaluate how often family doctors believed they should oversee the use of high-risk and low-risk medications yielded values of 3.38 for the former and 2.61 for the latter (p < 0.001). Only 24% responded correctly to all three items that inquired about the appropriateness of the advice they gave to older patients. On a scale of 1 to 4, the frequency at which family doctors gave older patients advice about preventive measures was 2.85, and only 43% reported allocating time during appointments to provide this advice. These latter two variables were directly associated with appropriate values for the index used to evaluate physicians’ oversight of medications associated with a high risk. The perception of risk associated with medications and involvement in preventive activities were both greater among female participants.

**Conclusions:**

Family doctors correctly identified medications according to their risk of playing a role in traffic accidents, although the recommendations they gave to their patients were not always appropriate. These findings, along with physicians’ infrequent involvement in preventive activities, suggest a need to improve family doctors’ competencies and increase the resources available to them so that they can provide their older patients with advice on ways to prevent involvement in traffic accidents.

**Supplementary Information:**

The online version contains supplementary material available at 10.1186/s12877-023-04316-z.

## Background

Morbidity and mortality associated with traffic accidents (TA) is a health problem of particular relevance for older populations. In 2020, although people older than 65 years represented only 16.5% of all drivers in Spain [1], they accounted for 26% of all deaths due to TA [2]. Moreover, this problem is likely to worsen substantially in the near future because of population aging and increasing mobility among older people [3–6]. One of the factors associated with increased risk of involvement in a TA among older drivers is their frequent use of medications that curtail the neurosensory and cognitive capacities needed for safe driving [7–14].

In Spain, family medicine has been a medical specialty since 1978. Family doctors (FD) who work for the Public Health System (PHS) do so mainly in both Emergency Services (hospital or out-of-hospital) and Primary Health Care Centers (PHCC). These centers constitute the gateway to the first level of care of the health system in our country and attends on average more than 250 million consultations annually, mostly from patients over 65 years of age with chronic pathologies and polymedicated patients [15]. Family doctors (FD) play an essential role in identifying and reducing this risk factor [16–19]. First, FD are the healthcare actors who usually prescribe potentially risky medications for their older patients, and are thus the care providers who should be familiar with the association between these drugs and the likelihood of involvement in a TA – not only in the general population but especially in older patients. In the latter group, both multiple medication use and interactions with other risky circumstances are higher than average [7, 8, 11, 14, 20–22]. Second, almost all persons older than 65 years regularly contact their primary healthcare provider, and it is at this level of care where most of their health problems are managed. However, few studies of FD or of older patients have focused on factors related with medication use and TA. The relatively few studies published to date have centred on how frequently FD ask their older patients about the use of medications that might affect their driving, and on whether FD offer these patients advice regarding the risk of driving while using these drugs [22–26]. In Spain, unfortunately, there appear to be no studies of the association between medications and the risk of TA among older drivers. Similarly, little research is available on the levels of knowledge of FD regarding medications related with the likelihood of involvement in a TA and the appropriateness of the advice on medication use and driving that they should give their older patients. We located only one study, published in 2001, of a sample of 187 healthcare professionals (87 of whom were FD) at health centres in Barcelona. The participants in this study completed a test of their knowledge of TA that included three questions about medications [27], but did not specifically investigate how they dealt with driving by their older patients.

Given the lack of information about this topic despite its relevance – we feel – to public health, we designed the present study with the aim of evaluating the knowledge of FD in Spain concerning the medications they prescribe most frequently for their older patients which may increase these patients’ risk of involvement in a TA. Additional aims were to document the level of involvement of FD in preventive activities linked to these prescriptions, and to determine the association between these factors and specific demographic and workplace characteristics of this group of primary care physicians.

## Methods

The data collection of this cross-sectional study was carried out in Spain from October 2016 to October 2018, and the data were from the responses on a self-administered questionnaire. Details of the study and questionnaire design and the process of questionnaire validation were published previously. The objective of said questionnaire was to evaluate the knowledge, attitudes and strategies for the prevention of traffic accidents in elderly drivers carried out by FD. The knowledge and strategies evaluated specifically include those referring to the consumption of drugs that may increase the risk of suffering a TA [28]. Briefly, the target population consisted of all FD working in Spain at primary healthcare centres or emergency services. The sample was recruited with two complementary strategies. (1) All FD who were members of at least one of the three main professional associations for family and community medicine (SemFyC), primary care medicine (SEMERGEN) and general and family medicine (SEMG) were contacted by email to solicit their participation in the study and invite them to complete an online version of the questionnaire. (2) A printed version of the questionnaire was distributed to FD who attended the main primary healthcare conferences held in Spain during the study period. These two strategies together yielded a total of 1897 returned questionnaires. Because of missing data for the autonomous region of residence, 9 questionnaires were excluded, for a final sample of 1888 FD.

This study was approved by the Hospital Universitario Clínico San Cecilio Ethics Committee (Reference Code: 1451-N-17.I.P.). All participants received information regarding the study aims, and signed an informed consent form before completing the questionnaire. To ensure confidentiality of the data, all participants were anonymized through a code accessible only to the researchers. Informed consent information and personal data were stored separately.

The questionnaire (Additional file 1) included items that aimed to evaluate three constructs: attitudes of FD toward TA prevention in older people, usual practices during appointments in connection with this health issue, and their level of knowledge about different aspects of this issue. The factors, variables and associations analysed in the present study are described below.

1) Knowledge about medications that can increase the risk of TA. Participants were asked to indicate, on a Likert-like scale, how often they believed they should oversee the use (at standard doses) of 11 different groups of drugs in order to prevent the risk of TA in drivers aged 65 years or older. The response options were 1 (never or hardly ever), 2 (sometimes), 3 (often) and 4 (always). Originally, these 11 groups pertained to two categories defined a priori on the basis of our literature review: drugs associated with a high risk of interfering with fitness to drive (antiepileptics, antihistamines, benzodiazepines and muscle relaxants, codeine-based cough suppressants, and opioid analgesics), and those not associated with a high risk of interfering with driving ability (antiplatelet agents, antianginal agents, beta blockers, insulin and/or oral antidiabetics, angiotensin-converting enzyme inhibitors [ACEIs] and nonsteroidal anti-inflammatory drugs [NSAIDs]) [12, 21, 29–34]. During the process of questionnaire validation [28], factor analysis of the items in this part of the questionnaire disclosed that when the “insulin and/or oral antidiabetics” group was excluded, the remaining drugs or groups of medications were distributed in two factors that corresponded to the two groups defined a priori. For each group an average index was obtained by dividing the sum of the scores for all items in each group by 5: low-risk drug index (LRDI) and high-risk drug index (HRDI). In addition, the questionnaire contained three items that asked FD to choose which of three types of advice about driving was the most appropriate for older patients who had begun treatment with antidepressants, antipsychotics, or antacids/proton pump inhibitors. Each correct response was scored as 1 point and the other two (incorrect) options were scored as 0 points. The scores on these three items were used to construct the “number of correct responses” (NCR) variable with a value of 0–1, 2, or 3.

2) Actions to prevent TA in older drivers who use different medications. The respondents’ preventive activities were investigated in two measures. The first was a polytomous response index (PRI) based on a series of 14 types of advice with Likert-like response options that asked FD to indicate how often they gave their older patients each type of advice about preventing TA. The advice specifically concerning medication use was worded as “Don’t take medicines without consulting your doctor or your pharmacist”. The response options were 1 (never or hardly ever), 2 (sometimes), 3 (often), or 4 (always). The second measure was a dichotomous response index (DRI) based on a series of six items that asked FD whether they carried out different preventive activities. The item concerning medication use asked, “Do you give older adults any preventive advice about traffic accidents according to their health problems and medications?”. The inclusion of the items from Sects. 1 and 2 as well as the correct meaning or not of the answers that the FD offered to such questions was based on the two aspects mentioned above: the review of the literature [12, 21, 29–34] and the agreement of their answers respect to the subscales extracted from the questionnaire validation process: drugs whose consumption implied a low or high risk of being involved in TA [28].

3) Sociodemographic variables and variables related to workplace characteristics. This part of the questionnaire solicited information on the autonomous region of residence, age, gender, nationality, and professional status (resident, specialist). Participants were also asked to note the type of centre where they worked (health centre, auxiliary health centre, emergency service, private practice, or other), location of the centre (urban or rural), years of experience, approximate number of patients on their roster, and approximate percentage of patients older than 65 years on their roster (less than 20%, between 20% and 39%, between 40% and 59%, 60% or more).

### Analysis

Descriptive parameters were first estimated for all medication-related variables. Because the distribution of gender and autonomous region of residence in our sample was not representative of the values estimated for the entire FD population working in Spain, the values for all estimators were weighted by the ratio of proportion of FD observed in our sample for each region and gender to the corresponding value estimated for the whole country, according to data provided by the Ministry of Health [35].

The next step was to determine the magnitude of association between each of the three variables for knowledge about medications (LRD, HRD, and NCR) and the two indexes of preventive activities (DRI and PRI). For DRI (used as a dependent variable) a conventional logistic regression model was used, and for PRI an ordinal logistic regression model was used. Both models were used to calculate the odds ratio (OR) as a measure of association. In the ordinal model, the estimate assumed a constant value for fold increase in the odds between a given outcome and the next highest one (on a scale of 1 to 4 possible outcomes) per unit increase in the independent variable. For all estimates, the 95% confidence interval (95% CI) was calculated.

In the final step, multivariate regression models were constructed to estimate the adjusted association for FD demographic and workplace variables with each of the medication-related variables (knowledge and attitudes toward prevention). Linear regression models were used for LRDI and HRDI, ordinal logistic regression models were used for NRC and PRI, and conventional logistic regression models were used for DRI. In the linear models the regression coefficient was calculated for each independent variable, and in the logistic models the OR (and its 95% CI) was calculated. Models were first fitted for the entire sample with FD gender, age, nationality and region of residence. Then additional models were constructed for specialist FD; these models included the variables specified above and also all remaining variables pertaining to professional activity. All analyses were done with the Stata statistical package (v. 16.0) [36].

## Results

Additional file 2 shows how our sample was distributed for all study variables. Table 1 presents the mean weighted values for gender and region of residence according to participants’ scores on how often they believed they should monitor their patients’ use of each drug or group of drugs. Indexes for all medications considered a priori to be related with a high risk of TA ranged from 3.13 (codeine-based cough suppressants) to 3.64 (benzodiazepines). For HRDI the mean value was 3.38 (equivalent to “often” and “always” responses). For medications related with a low risk of TA, indexes were significantly lower, with a mean of 2.61 for LRDI (equivalent to the mean frequency of “sometimes” and “often” responses). The mean index was higher among female FD (2.71) than male FD (2.48). The difference between indexes for HRDI and LRDI was statistically significant (Student’s t test for paired samples: -42.58; p < 0.001).

**Table 1:** Mean weighted values of scores for each drug or group of drugs in the item “Indicate how often you think that family doctors should record the use of the following types of medication in the medical record (standard dose) in order to lower the risk of involvement in traffic accidents in drivers who are more than 65 years old” (1: Never or hardly ever; 2: Sometimes; 3: Often; 4: Always)

Drug or Index	Total			Women			Men		
	N^1^	Mean	95% CI	N^1^	Mean	95% CI	N^1^	Mean	95% CI
Antiepileptics	1682	3.46	3.41–3.52	1140	3.47	3.41–3.53	542	3.46	3.37–3.55
Antihistamines	1681	3.14	3.07–3.21	1140	3.21	3.14–3.28	541	3.05	2.91–3.18
Benzodiazepines	1680	3.64	3.59–3.68	1137	3.62	3.57–3.68	543	3.66	3.59–3.73
Codeine-based cought suppressants	1682	3.13	3.07–3.20	1139	3.23	3.16–3.30	543	3.02	2.90–3.13
Opioid analgesics	1685	3.48	3.43–3.54	1141	3.51	3.45–3.57	544	3.45	3.36–3.55
*HRDI*	1669	3.38	3.33–3.42	1131	3.41	3.36–3.46	538	3.34	3.27–3.41
Antiplatelet agents	1683	2.56	2.49–2.64	1140	2.69	2.61–2.74	543	2.41	2.28–2.54
Antianginal agents	1683	2.89	2.82–2.95	1142	2.96	2.89–3.03	541	2.79	2.68–2.90
Beta blockers	1682	2.99	2.92–3.05	1141	3.04	2.98–3.11	541	2.92	2.80–3.03
ACEIs	1682	2.43	2.37–2.49	1140	2.58	2.51–2.65	542	2.25	2.16–2.35
NSAIDs	1682	2.17	2.10–2.23	1141	2.30	2.22–2.37	541	2.01	1.91–2.11
*LRDI*	1669	2.61	2.56–2.66	1136	2.71	2.65–2.77	533	2.48	2.39–2.56

1 N: Number of valid responses for each item or index

Table 2 summarizes the distribution of participants’ responses to items about appropriate advice for drivers older than 65 years who had started to use different types of medications. For antidepressants, 75% of FD chose the correct response (Don’t drive during the first few days). For antacids, 76% of FD chose the correct response (Can continue driving). However, for antipsychotics, the correct response (Don’t drive) was chosen by only 46% of participants; the response chosen most frequently was “Don’t drive during the first few days” (49%). Overall, only 24% of FD responded correctly on all three items. There were no substantial differences in the percent of correct responses between genders.

**Table 2:** Distribution of FD responses on items about appropriate recommendations for drivers older than 65 years who had started using different types of medication

Type of drug and number of valid responses	Response options	Total		Women		Men	
		P^1^	95% CI	P^2^	95% CI	P^2^	95% CI
Antidepressants Total:1688 Women: 1143 Men: 545	Don’t drive	0.09	0.08–0.11	0.11	0.08–0.13	0.08	0.05–0.11
	Don’t drive during the first few days (correct response)	0.75	0.72–0.78	0.72	0.69–0.76	0.78	0.73–0.84
	Can continue driving	0.16	0.13–0.19	0.17	0.13–0.20	0.14	0.09–0.19
Antipsychotics Total: 1686 Women: 1140 Men: 546	Don’t drive (correct response)	0.46	0.42–0.50	0.45	0.41–0.49	0.47	0.40–0.54
	Don’t drive during the first few days	0.49	0.45–0.53	0.49	0.45–0.53	0.49	0.42–0.56
	Can continue driving	0.05	0.03–0.07	0.05	0.03–0.07	0.04	0.01–0.08
Antacids or proton pump inhibitors Total: 1689 Women: 1143 Men: 546	Don’t drive	0.04	0.02–0.05	0.04	0.02–0.05	0.03	0.01–0.05
	Don’t drive during the first few days	0.20	0.18–0.23	0.19	0.16–0.21	0.23	0.18–0.27
	Can continue driving (correct response)	0.76	0.73–0.79	0.78	0.75–0.81	0.74	0.69–0.80
Number of correct responses Total: 1684 Women: 1139 Men: 545	0–1	0.26	0.23–0.29	0.28	0.24–0.31	0.24	0.19–0.30
	2	0.50	0.46–0.53	0.49	0.45–0.53	0.50	0.43–0.57
	3	0.24	0.21–0.28	0.23	0.19–0.26	0.26	0.19–0.32

1 Estimates weighted by the distribution of FD by gender and region of residence in the reference population

2 Estimates weighted by the distribution of FD by region of residence in the reference population

Analysis of the responses regarding preventive recommendations offered by FD yielded a mean score of 2.85 (95% CI: 2.77–2.94) for PRI, which was very close to the value for the “often” option, and no difference between genders was observed. However, only 43% of FD (95% CI: 0.39–0.47) chose the “Yes” response for DRI, and this value was higher in male FD (47%) than in female FD (39%).

Table 3 shows the associations between each of the three variables used to explore level of knowledge about different medications and the frequencies of engaging in preventive activities. For DRI, only HRDI yielded a direct association, with an OR de 1.56. For PRI, all three variables showed a positive association, although the greatest strength of association was again found for HRDI (OR = 1.39).

**Table 3:** Logistic regression models to quantify the association of level of knowledge about medications and their influence on older patients’ likelihood of involvement in a traffic accident with the frequency of preventive activities

	Conventional logistic regression. Dependent variable: DRI (0: No/1: Yes) (n = 1642)				Ordinal logistic regression. Dependent variable: PRI (1: Never or hardly ever; 2: Sometimes; 3: Often; 4: Always) (n = 1640)			
Independent variable	Odds ratio^1^	95% CI		p	Odds ratio^1,2^	95% CI		p
*LRDI*	0.91	0.79	1.05	0.211	1.24	1.09	1.42	0.002
*HRDI*	1.56	1.29	1.89	< 0.001	1.39	1.18	1.64	< 0.001
*NCR*	0.92	0.80	1.06	0.275	1.13	1.00	1.28	0.055

1 All odds ratio estimates adjusted for the remaining independent variables shown for each model

2 Odds ratio values estimated with ordinal logistic regression models were given a constant value for fold increase in the odds between one outcome and the following outcome (on a scale of 1 to 4 possible outcomes) per unit increase in the independent variable

Table 4 presents the results of each multivariate model used to estimate adjusted associations of demographic and workplace characteristics of FD with HRDI and LRDI. In all models, male gender was associated with lower values for both indexes. In models built for the entire sample of FD, older age was associated with lower LRDI values and with higher HRDI values. However, both of these associations were absent in the model used to analyze the results for specialist primary healthcare providers separately. In this subgroup the only association of note was the decrease in LRDI together with the decrease in the proportion of patients 65 years or older on their roster. The NCR variable was not associated with any of the FD demographic or workplace variables, as detailed in Additional file 3.

**Table 4:** Strength of association of LRDI and HRDI with demographic and workplace characteristics of family doctors. Multiple linear regression models

	Dependent variable: LRDI								Dependent variable: HRDI							
	Entire sample (n = 1664)				Specialists (n = 54)				Entire sample (n = 1663)				Specialists (n = 757)			
Variable	Coef.^1^	95% CI		P	Coef.^1^	95% CI		P	Coef.^1^	95% CI		P	Coef.^1^	95% CI		P
Gender (Ref: Female)																
Male	-0.21	-0.29	-0.13	< 0.001	-0.28	-0.39	-0.16	< 0.001	-0.12	-0.18	-0.06	< 0.001	-0.13	-0.23	-0.04	0.004
Age																
Per 10-year group	-0.07	-0.10	-0.04	< 0.001	-0.05	-0.17	0.08	0.462	0.03	0.01	0.06	0.008	0.01	-0.09	0.11	0.838
Nationality (Ref. Spanish)																
Other	0.13	-0.04	0.31	0.140	0.24	-0.16	0.64	0.241	0.11	-0.03	0.25	0.133	0.15	-0.16	0.46	0.338
Experience																
Per 10-year group					0.04	-0.08	0.16	0.508					0.03	-0.07	0.12	0.570
Number of patients on roster (Ref: 1000 or fewer)																
1001 to 1500					0.00	-0.19	0.20	0.960					-0.05	-0.20	0.10	0.509
More than 1500					0.05	-0.15	0.25	0.654					-0.01	-0.16	0.15	0.947
Proportion of patients older than 65 years on roster (Ref: 60% or more)																
45–59%					-0.20	-0.40	-0.01	0.043					-0.10	-0.25	0.06	0.208
20–39%					-0.24	-0.43	-0.05	0.015					-0.10	-0.25	0.05	0.173
< 20%					-0.42	-0.66	-0.18	0.001					-0.18	-0.37	0.01	0.065
Type of center (Ref: Health center)																
Auxiliary health center					0.03	-0.20	0.25	0.815					0.08	-0.09	0.26	0.351
Emergency service					0.10	-0.35	0.54	0.666					-0.07	-0.41	0.28	0.688
Other					0.46	-0.02	0.94	0.058					0.18	-0.19	0.56	0.336
Location of center (Ref: Urban)																
Rural					0.04	-0.09	0.16	0.566					0.02	-0.07	0.12	0.623

1 All regression coefficient estimates were adjusted for the remaining variables shown for each model and for region of residence. Coefficients for which the 95% CI did not include 0 are shaded

Lastly, Table 5 summarizes the results from multivariate models that used DRI or PRI as the dependent variable. Models with DRI yielded a direct association only for FD age, and this association was absent when the analysis was restricted to the subgroup of specialists. In models with PRI, male gender was apparently associated with a lower mean index. In addition, FD with fewer than 20% of patients older than 65 years on their roster also had a significantly lower mean index.

**Table 5:** Strength of association of DRI and PRI with demographic and workplace characteristics of family doctors. Logistic regression models

	Conventional logistic regression Dependent variable: DRI								Ordinal logistic regression Dependent variable: PRI							
	Entire sample (n = 1769)				Specialists (n = 811)				Entire sample (n = 1768)				Specialists (n = 812)			
Variable	OR^1,2^	95% CI		P	OR^1,2^	95% CI		P	OR^2,3^	95% CI		P	OR^1,2,3^	95% CI		P
Gender (Ref: Female)																
Male	1.01	0.82	1.25	0.929	1.01	0.74	1.37	0.949	0.84	0.70	1.02	0.073	0.76	0.58	1.00	0.049
Age																
Per 10-year group	1.16	1.07	1.25	< 0.001	0.97	0.70	1.34	0.853	0.98	0.91	1.06	0.638	0.91	0.68	1.20	0.491
Nationality																
Other	1.26	0.78	2.03	0.348	0.67	0.21	2.10	0.494	1.20	0.78	1.86	0.404	1.49	0.54	4.06	0.441
Years of experience (Ref: <5)																
Per 10-year group					1.16	0.85	1.58	0.349					1.24	0.95	1.62	0.119
Number of patients on roster (Ref: 1000 or fewer)																
1001 to 1500					0.72	0.43	1.21	0.224					0.75	0.48	1.19	0.225
More than 1500					1.04	0.61	1.78	0.884					1.08	0.67	1.74	0.750
Proportion of patients older than 65 years on roster (Re: 60% or more)																
45–59%					1.39	0.82	2.36	0.224					1.01	0.62	1.67	0.940
20–39%					1.24	0.74	2.08	0.416					0.68	0.42	1.10	0.116
< 20%					0.68	0.35	1.34	0.266					0.39	0.21	0.72	0.002
Type of center (Ref: Health center)																
Auxiliary health center					0.86	0.47	1.59	0.635					0.82	0.49	1.39	0.464
Emergency service					1.53	0.45	5.17	0.497					1.84	0.61	5.57	0.282
Other					0.68	0.18	2.61	0.576					0.86	0.25	2.97	0.815
Location of center (Ref: Urban)																
Rural					1.25	0.89	1.75	0.198					0.87	0.64	1.18	0.372

1 OR: odds ratio

2 All OR estimates were adjusted for the remaining variables shown for each model and for region of residence

3 Odds ratio values estimated with ordinal logistic regression models were given a constant value for fold increase in the odds between a given outcome and the next highest one (on a scale of 1 to 4 possible outcomes) per unit increase in the independent variable

Estimated OR values for which the 95% CI did not include 1 are shaded

## Discussion

The most relevant findings of this study are discussed below along with their implications and our suggestions for strategies FD could use to reduce the risk of TA among older drivers.

1. Family doctors clearly distinguished between medications that increased the risk of causing TA and those with no substantial influence on this risk. However, the overall frequency of the view that they should monitor the use of high-risk drugs was lower than desirable at 3.38 out of 4 (equivalent to the “always” response). Moreover, knowledge of the relation between specific drugs and TA risk did not imply that FD consistently provided appropriate recommendations to their older patients. Only 24% of participants gave their older patients appropriate advice regarding their fitness to drive. Of note, almost 50% of FD indicated they would allow patients to drive a few days after starting to use an antipsychotic drug, although the use of these agents makes it advisable to stop driving entirely. A notable parallel finding was that only drugs associated with a low level of risk were associated with the perception by FD that the use of these drugs by older patients should be monitored, and with a larger proportion of older patients on their roster.

Because of the lack of similar studies, it is challenging to compare our findings with earlier research. In Spain, a study by Martín Cantera [27] (see Background) reported correct response rates of 84.0%, 70.6% and 26.2% for three items that explored physicians’ knowledge and centered on the association between medications and involvement in TA. (Specifically, these items explored which prescription medications were least advisable for a truck driver who sought medical care for back pain, which antihypertensive drugs had the least effect on driving, and the most appropriate advice for a driver who had been prescribed antihistamines.) The lack of data notwithstanding, findings published to date suggest that FD should become more aware of the importance of overseeing the use of medications that may increase the risk of TA, and should strengthen their competencies in this area in order to provide better advice to their older patients.

2. The frequency with which FD in this study reported providing preventive advice to their older patients about the association of certain medications with the risk of TA can be considered too low. Although the overall frequency of providing such advice almost reached the category of “often”, only 43% indicated that they spent time during appointments actually providing this advice. Other studies have also found that physicians rarely discussed driving or TA risks with their older patients [37]. For example, a 2019 study by Betz et al. noted that only 29% of older people included in the LongROAD study cohort had had conversations with their doctor about medication use, driving, and related topics [26]. However, a study in Switzerland by Sebo et al. found that 96% of all FD often or always asked their older patients who drove about the list of their current medications, 88% inquired about their use of antidiabetic drugs, and 65.5% asked patients about their use of psychotropic drugs [25]. In Canada, Jang et al. reported that 89% of FD often or always reviewed the medications prescribed for their older patients who drove [23]. These data suggest that FDs in other countries are more involved in providing advice to their patients about medications and driving than FD in Spain. The findings again suggest a need to increase the resources available to FD in this country and raise their awareness of potential risks in order to strengthen their involvement in preventive activities aimed at reducing TA risks in older drivers. In addition, we observed a direct association between the ability to identify high-risk drugs more accurately and a higher frequency of involvement in preventive actions intended to reduce the risk of TA in patients for whom these drugs were prescribed. This finding provides further evidence that enhancing family physicians’ knowledge about the appropriate management of prescription medications is likely to be effective in reducing the risk of causing TA in older drivers.

3. Female gender was the only FD characteristic that was apparently associated with a greater perceived risk linked to the use of some medications, and with greater involvement in preventive activities for patients who were prescribed these medications. This finding is consistent we earlier studies: compared to their male counterparts, female physicians and FD gave their patients more information and spent more time on each appointment [9, 27, 31]. Research in Switzerland also found that female general practitioners used a traffic medicine website more frequently than their male counterparts [24]. These results are consistent with a culture that assumes women to be the main caregivers for older people. It would be interesting to further investigate the reasons that underlie gender-related differences identified in the present study.

### Strenghts and limitations

Aside from the cross-sectional nature of this study, limitations in two other main areas should be noted: the validity of the instrument used to collect information, and the representativeness of the sample of FD. Regarding validity, all potential information biases that can arise with the use of a self-administered questionnaire may be present. In this connection it is worth noting that attempts to determine the level of knowledge among FD about our research topic are potentially sensitive, given that no professional group enjoys being subjected to an evaluation of their knowledge and competencies. By framing the relevant item in the Accident Risk and Medication section as a way to obtain information on “how often you think that family doctors should record the use of the following types of medication”, our aim was to obtain an indirect measure of the level of knowledge among FD and thus avoid the implication that they were being tested in this area. In addition, information bias arising from the participants’ desire to meet the questionnaire authors’ expectations may have resulted in overestimation of the scores in this item, as well as overestimation of the frequencies of involvement in different preventive actions.

Because participation in this study was voluntary and given the strategies used to recruit respondents, we are aware that our sample cannot be considered representative of all FD who work in Spain, although weighting by gender and autonomous region of residence may have palliated the overrepresentation of women and FD working in the region of Andalusia. In consonance with the considerations noted above, we assume that FD who chose to participate in the study were more highly motivated to contribute to research on this topic – a source of bias that may have led to overestimation of the values for variables that explored involvement in preventive activities. Despite these limitations, potential merits of our design and analysis worth emphasizing are the large sample size and participation of FD from all autonomous regions in the country, and the use of a questionnaire that was suitably validated before use in the target population [28].

## Conclusions

The results strongly suggest that although FD working in Spain are able to identify which of the medications they prescribe for their older patients are related with a risk of causing TA, their knowledge does not always translate into appropriate recommendations for these patients. This situation, together with the limited involvement of FD in actions aimed at preventing TA, makes it necessary to strengthen their competencies in managing their practices when they prescribe drugs that may increase the risk of TA in older drivers. In addition, FD should be equipped with additional resources to facilitate implementation of their competencies in situations where their older patients would benefit from receiving more, better advice regarding ways to prevent traffic accidents when they use certain types of medications.

### Electronic supplementary material

Below is the link to the electronic supplementary material.

Supplementary Material 1

## Data Availability

The datasets used for this study are available upon reasonable request to the corresponding author. All the methods, materials and original questionnaire used for this work are included in this manuscript and its supplementary files. This study was not pre-registered.

## Abbreviations

TA: Traffic accidents
FD: Family doctors
ACEIs: Angiotensin-converting enzyme inhibitors
NSAIDs: Nonsteroidal anti-inflammatory drugs
LRDI: Low-risk drug index
HRDI: High-risk drug index
NCR: Number of correct responses
PRI: Polytomous response index
DRI: Dichotomous response index
OR: Odds ratio
