# Supplementary material for: Medications and traffic accidents involving older drivers: do Spanish primary healthcare physicians know enough?
Source: BMC Geriatr. 2023 Oct 17;23:669. doi: 10.1186/s12877-023-04316-z (PMC10583376; doi:10.1186/s12877-023-04316-z)
Supplement: Supplementary file 1 — Supplementary Material 1 [file 12877_2023_4316_MOESM1_ESM.docx]

**Additional file 1.** Questionnaire on knowledge, attitudes and current practices of primary health care physicians regarding the prevention of road injuries in older adults (paper version).

**1. DEMOGRAPHIC AND WORKPLACE DATA**

- Date of completion: |_|__|--|__|__|--|__|__|
- Sex: Male □ Female □
- Age: ______
- Nationality: Spanish □ / Other □ (please specify): ________________________
- Where do you work?: _______________________________________________
- Province: _____________________________
- Are you an intern in Family and Community Medicine? Yes □ / No □.

If yes, please go to Part 2.

- Type of healthcare centre where you work:

Health centre □ / Auxiliary health centre □ / Emergency services □ /

Private practice □ / Other (specify): _________________________________

- Workplace location: Urban □ Rural □
- Approximate number of patients on your roster: ___________
- Choose the approximate percentage of patients older than 65 years who are on your roster: Less than 20% □ / Between 20% and 39% □ / Between 40% and 59% □ / 60% or more □
- How many years of experience do you have as a family doctor?

(If less than 1 year, please indicate “0”): |__|years.

**2. ATTITUDES AND ACTIVITIES**

**During your appointments, do you often ask older adults if they drive any motor vehicles?**

□ Yes □ No □ Don’t know / No answer

**Do you note their answer in their medical record?**

□ Yes □ No □ Don’t know / No answer

**Do you give older adults any preventive advice about traffic accidents according to their health problems and medications?**

□ Yes □ No □ Don’t know / Not answer

**Do you ask your older adult patients when they last renewed their driver’s license?**

□ Yes □ No □ Don’t know / No answer

**Have you prepared any individual health reports at the request of a Driver Examination Centre?**

□ Yes □ No □ Don’t know / No answer

**Do you use any guidelines to evaluate the ability of your older adult patients to drive?**

□ Yes □ No □ Don’t know / No answer

**Indicate how often you give each of the following preventive recommendations about traffic accidents to your older adult patients** (1: Never or hardly ever; 2: Sometimes; 3: Often; 4: Always).

| **Recommendations** | **1** | **2** | **3** | **4** |
| --- | --- | --- | --- | --- |
| - Drive on familiar roads in well-lit areas and avoid risky maneuvers |  |  |  |  |
| - Avoid heavy traffic and complicated routes |  |  |  |  |
| - Use public transport, especially for long journeys |  |  |  |  |
| - If possible, try not to travel alone |  |  |  |  |
| - Avoid driving at night and in bad weather |  |  |  |  |
| - Rest after every two hours of driving |  |  |  |  |
| - Avoid using the car if you don’t feel well, have had a bad night, or your doctor has prescribed a new treatment |  |  |  |  |
| - Don’t take medicines without consulting your doctor or your pharmacist |  |  |  |  |
| - Don`t drink alcohol if you have to drive |  |  |  |  |
| - When you go to specialists, remind them that you drive |  |  |  |  |
| - If you need glasses or use a hearing aid, get regular check-ups |  |  |  |  |
| - Don’t cross the road outside pedestrian crossings or if visibility is limited |  |  |  |  |
| - Avoid walking along roads that have no verge or sidewalk |  |  |  |  |
| - Avoid crossing the road while using your cell phone |  |  |  |  |

**Indicate your level of agreement with each of the following statements** (1: Disagree; 2: Neither agree nor disagree; 3: Partially agree; 4: Completely agree)**.**

| **Statements** | **1** | **2** | **3** | **4** |
| --- | --- | --- | --- | --- |
| - Family doctors should ask older adult patients if they drive motor vehicles |  |  |  |  |
| - Family doctors should gather information on older patients’ driving habits (e.g. type of vehicle, routes, reasons for driving, distance, driving alone or with a passenger) |  |  |  |  |
| - Family doctors should offer older adults health advice about preventing traffic accidents |  |  |  |  |
| - Family doctors should spend some time during appointments to identify older adult drivers at high risk of traffic accidents |  |  |  |  |
| - If an older adult’s relatives consider they cannot drive safely, they should get in touch with the older driver’s family doctor |  |  |  |  |
| - Family doctors should be legally qualified to submit reports that advise limiting an older patient’s legal permission to drive |  |  |  |  |
| - Family doctors should be responsible for driving license renewals for older adults |  |  |  |  |
| - Family doctors have a conflict of interest (confidentiality vs. public safety) regarding the provision of information to the authorities about at-risk drivers |  |  |  |  |
| - Family doctors should be aware that telling older adults that they are at-risk drivers can negatively affect their doctor–patient relationship |  |  |  |  |
| - Family doctors should be aware that suspension of an older adult’s driver’s license can negatively affect their health and well-being |  |  |  |  |
| - Family doctors would benefit from guidelines about the prevention of traffic injures in primary care |  |  |  |  |

**Rank the following factors according to how important you think they are for preventing accidents in older primary care patients** (1 = most important; 4 = least important):

| - Increase awareness of healthcare professionals |  |
| --- | --- |
| - More training in this area |  |
| - More time for appointments |  |
| - More resources (e.g. guidelines) |  |

**3. ACCIDENT RISK AND EXISTING HEALTH PROBLEMS**

**Indicate how often you think that family doctors should record the following health problems in the medical record in order to lower the risk of involvement in traffic accidents in drivers who are more than 65 years old (**1: Never or hardly ever; 2: Sometimes; 3: Often; 4: Always).

| **Health problems** | **1** | **2** | **3** | **4** |
| --- | --- | --- | --- | --- |
| - Progressive vision problems |  |  |  |  |
| - Mild hearing loss |  |  |  |  |
| - Hypertension controlled with medication |  |  |  |  |
| - Atrial fibrillation |  |  |  |  |
| - Diabetes mellitus |  |  |  |  |
| - Severe obesity |  |  |  |  |
| - Sleep apnea |  |  |  |  |
| - Migraine |  |  |  |  |
| - Peripheral vertigo |  |  |  |  |
| - Alcohol dependence |  |  |  |  |
| - Depression |  |  |  |  |
| - Muscle strength or tone disorders |  |  |  |  |

**What advice would you give to a driver older than 65 years who recently had an episode of ischemic heart disease and is now asymptomatic?**

□ Don’t drive □ Drive in a week

□ Drive in a month □ Drive in three months

**What advice would you give to a driver older than 65 years who has had deep vein thrombosis?**

□ Don’t drive □ Drive in a week

□ Drive in a month □ Drive in six months

**What advice would you give to a driver older than 65 years who has a personality disorder or early-stage dementia?**

□ Don’t drive □ Drive only in familiar areas

□ Drive only if you are accompanied □ Drive if you have no symptoms

**4. ACCIDENT RISK AND MEDICATIONS**

**Indicate how often you think that family doctors should record the use of the following types of medication in the medical record (standard dose) in order to lower the risk of involvement in traffic accidents in drivers who are more than 65 years old (**1: Never or hardly ever; 2: Sometimes; 3: Often; 4: Always).

| **Type of medication** | **1** | **2** | **3** | **4** |
| --- | --- | --- | --- | --- |
| - Antiplatelet agents |  |  |  |  |
| - Antianginal agents |  |  |  |  |
| - Beta blockers |  |  |  |  |
| - Antiepileptics |  |  |  |  |
| - Insulin and oral antidiabetics |  |  |  |  |
| - Angiotensin-converting enzyme inhibitors (ACEIs) |  |  |  |  |
| - Nonsteroidal antiinflammatory drugs (NSAIDs) |  |  |  |  |
| - H1 antihistamines |  |  |  |  |
| - Benzodiazepines and muscle relaxants |  |  |  |  |
| - Codeine-based cough suppressants |  |  |  |  |
| - Opioid analgesics |  |  |  |  |

**What advice would you give to a driver older than 65 years who has started to use the following medications?**

*Antidepressants*

□ Don’t drive □ Don’t drive during the first few days after starting a new medication □ Can continue driving

*Antipsychotics*

□ Don’t drive □ Don’t drive during the first few days after starting a new medication □ Can continue driving

*Antacids or proton pump inhibitors*

□ Don’t drive □ Don’t drive during the first few days after starting a new medication□ Can continue driving

**5. IMPORTANCE OF ACCIDENT RISK FOR TRAFFIC SAFETY IN OLDER ADULTS**

**On a scale of 1 (least important) to 10 (most important), where would you place…?**

- Traffic accidents as a public health problem in older adults |__|
- The **relevance that family doctors should have** in preventing traffic accidents in older adults|__|
- The **relevance that family doctors have now** in preventing traffic accidents in older adults |__|

**Rank the following four age groups according to the magnitude of their traffic accident death rate (1 = group with the highest rate, 4 = group with the lowest rate):**

| - 15-24 years |  |
| --- | --- |
| - 25-34 years |  |
| - 45-54 years |  |
| - 55-74 years |  |

**When you classify deaths from traffic accidents in older adults according to the type of road user, which type of user do you think accounts for the most deaths?**

□ Drivers □ Passengers □ Pedestrians □ Don’t know / No answer

**Additional file 2**. Distribution of the sample of family doctors according to demographic and professional characteristics.

| Variable | Category | N | %^1^ | %^2^ |
| --- | --- | --- | --- | --- |
| Gender | Female | 1283 | 67.96 | 54.6 |
|  | Male | 605 | 32.04 | 45.4 |
| Age group (years) | <30 | 608 | 32.20 | 32.76 |
|  | 30 to 34 | 211 | 11.18 | 13.49 |
|  | 35 to 39 | 160 | 8.47 | 7.52 |
|  | 40 to 44 | 152 | 8.05 | 7.04 |
|  | 45 to 49 | 164 | 8.69 | 9.83 |
|  | 50 to 54 | 191 | 10.12 | 8.31 |
|  | 55 to 59 | 252 | 13.35 | 12.79 |
|  | 60 or over | 142 | 7.52 | 8.26 |
|  | Unknown | 8 | 0.42 |  |
| Nationality | Spanish | 1808 | 95.76 | 94.65 |
|  | Other | 80 | 4.24 | 5.35 |
| Status | Specialist | 1123 | 59.48 | 55.99 |
|  | Resident | 758 | 40.15 | 44.01 |
|  | Unknown | 7 | 0.37 |  |
| Years of experience^3^ | <5 | 119 | 10.53 | 10.92 |
|  | 5 to 9 | 139 | 12.30 | 12.23 |
|  | 10 to 14 | 134 | 11.86 | 9.54 |
|  | 15 to 19 | 162 | 14.34 | 17.04 |
|  | 20 to 24 | 162 | 14.34 | 13.00 |
|  | 25 to 29 | 170 | 15.04 | 15.23 |
|  | 30 to 34 | 160 | 14.16 | 15.55 |
|  | 35 or more | 62 | 5.49 | 6.48 |
|  | Unknown | 22 | 1.95 |  |
| Number of patients on roster | 1000 | 121 | 10.71 | 10.77 |
|  | 1001 to 1500 | 399 | 35.31 | 42.29 |
|  | More than 1500 | 386 | 34.16 | 46.94 |
|  | Unknown | 224 | 19.82 |  |
| Proportion of patients older than 65 years on roster | 60% or more | 131 | 11.59 | 12.60 |
|  | 45% to 59% | 284 | 25.13 | 29.33 |
|  | 20% to 39% | 454 | 40.18 | 46.67 |
|  | <20% | 96 | 8.5 | 11.40 |
|  | Unknown | 165 | 14.6 |  |
| Type of centre^3^ | Health centre | 843 | 74.6 | 77.48 |
|  | Auxiliary health centre | 92 | 8.14 | 6.23 |
|  | Emergency service | 122 | 10.8 | 9.09 |
|  | Other | 62 | 5.49 | 7.20 |
|  | Unknown | 11 | 0.97 |  |
| Location of centre^3^ | Urban | 714 | 63.19 | 71.98 |
|  | Rural | 400 | 35.4 | 28.02 |
|  | Unknown | 16 | 1.42 |  |

1 Referred to original sample including missing values.

2 Referred to weighted sample (see Methods) excluding missing values.

3 Estimated only for the 1130 participants who were not residents.

**Additional file 3**. Strength of association of NCR with demographic and professional characteristics of family doctors. Ordinal logistic regression models.

|  |  | | | | | | | |
| --- | --- | --- | --- | --- | --- | --- | --- | --- |
|  | Entire sample (n=1678) | | | | Specialists (n=757) | | | |
|  | OR^1^ | 95% CI | | P | OR^1^ | 95% CI | | P |
| Males | 1.03 | 0.84 | 1.27 | 0.742 | 1.21 | 0.90 | 1.64 | 0.195 |
| Age | | | |  |  |  |  |  |
| Per 10-year group | 0.96 | 0.89 | 1.04 | 0.301 | 1.01 | 0.74 | 1.38 | 0.941 |
| Nationality (Ref. Spanish) | | | | |  |  |  |  |
| Other | 1.04 | 0.66 | 1.62 | 0.872 | 2.26 | 0.86 | 5.96 | 0.099 |
| Years of experience | | | | |  |  |  |  |
| Per 10-year group |  |  |  |  | 0.97 | 0.71 | 1.30 | 0.824 |
| Number of patients on roster (Ref: 1000 or more) | | | | | | | | |
| 1001 to 1500 |  |  |  |  | 1.21 | 0.74 | 1.96 | 0.452 |
| More than 1500 |  |  |  |  | 1.16 | 0.70 | 1.93 | 0.560 |
| Proportion of patients older than 65 years on roster (Ref: 60% or more) | | | | | | |  |  |
| 45% to 59% |  |  |  |  | 1.03 | 0.63 | 1.69 | 0.880 |
| 20% to 39% |  |  |  |  | 1.15 | 0.72 | 1.85 | 0.565 |
| <20% |  |  |  |  | 0.83 | 0.45 | 1.54 | 0.560 |
| Type of centre (Ref: Health centre) | | | | | | | | |
| Auxiliary health centre |  |  |  |  | 0.95 | 0.53 | 1.72 | 0.873 |
| Emergency service |  |  |  |  | 0.84 | 0.28 | 2.54 | 0.759 |
| Other |  |  |  |  | 2.15 | 0.68 | 6.82 | 0.193 |
| Location of centre (Ref: Urban) | | | | | |  |  |  |
| Rural | |  |  |  | 1.17 | 0.84 | 1.62 | 0.349 |

1 OR: Odds ratio. All OR estimates were adjusted for the remaining variables shown for each model and for region of residence. Odds ratio values were given a constant value for fold increase in the odds between a given outcome and the next highest one (on a scale of 1 to 3 possible outcomes) per unit increase in the independent variable.
